# Genome-Wide Identification, Gene Duplication, and Expression Pattern of *NPC2* Gene Family in *Parnassius glacialis*

**DOI:** 10.3390/genes16030249

**Published:** 2025-02-21

**Authors:** Zhenyao Zhu, Chengyong Su, Xuejie Guo, Youjie Zhao, Ruie Nie, Bo He, Jiasheng Hao

**Affiliations:** College of Life Sciences, Anhui Normal University, Wuhu 241000, China; m18555330464@163.com (Z.Z.); sky475342@163.com (C.S.); 15855714053@163.com (X.G.); bioala@ahnu.edu.cn (Y.Z.); neire2021@ahnu.edu.cn (R.N.); hebo90@126.com (B.H.)

**Keywords:** *NPC2* gene family, *Parnassius glacialis*, genome-wide analysis, gene duplication, transposable elements

## Abstract

Background: The Niemann–Pick C2 (*NPC2*) gene family plays an important role in olfactory communication, immune response, and the recognition of host plants associated with environmental adaptation for insects. Methods: In this study, we conducted a genomic analysis of the structural characteristics and physicochemical properties of the *NPC2* genes of eleven butterfly species available, focusing on the alpine *Parnassius* species, especially *Parnassius glacialis*, to investigate their duplication and expression patterns. Results: Our results indicate that a significant expansion of *NPC2* genes was detected in *P. glacialis* compared to other butterflies; in addition, the expansion of these unevenly distributed *P. glacialis NPC2* chromosome genes was shaped by tandem duplication mediated by transposons. Furthermore, the *PgNPC2* genes had relatively higher expression in *P. glacialis* antennae and other head tissues. These facts were verified by quantitative real-time PCR (qRT-PCR). Conclusions: These findings suggest that the expansion of *NPC2* genes may have contributed to the local adaptation of *P. glacialis* during its dispersal ‘out of the Qinghai–Tibet Plateau’, although further functional tests are needed to confirm their specific role in this adaptive process.

## 1. Introduction

The biological diversity pattern, especially in the Northern Hemisphere, has been significantly influenced by the geological events and climate changes in the Qinghai–Tibet Plateau (QTP) since the early Cenozoic era [1,2,3]. Among insect groups that originated on the QTP, the genus *Parnassius* is a typical high mountain-adapted butterfly group that is mainly distributed across the Holarctic region, especially on the QTP and neighboring mountains with an elevation ranging from about 3000 m to 5000 m. Previous studies have shown that the *Parnassius* probably originated in the early to middle Miocene about 20 to 15 million years ago (mya), and since that period, they have undergone rapid adaptive radiations and local specializations due to topographical isolation and habitat fragmentation events caused by the QTP uplifting, as well as climate changes, especially the glacial–interglacial cycles in the Quaternary Period [1,2,3]. Thus, this typical alpine butterfly group has emerged as an ideal model for studying the relationship between biological evolution and the earth’s environmental change, such as phylogenetics, phylogeography, and other areas of the earth–life system [4,5,6].

*P. glacialis* is the only *Parnassius* species that dispersed eastwards from the QTP to southern China (south of Yangtze River) and inhabits areas at altitudes ranging from about 2800 m to 300 m. Based on the genomic analysis of previous studies, this species began to disperse at about 1.1 to 0.6 mya, which coincides closely with the Kunlun-Huanghe tectonic movement [4,5,6,7], and during its dispersal processes, it underwent a rapid and extensive expansion of gene families (about 700), especially the *RPLP2* gene family, which contains about 434 genes [4,8,9]. Except for one gene that is structurally and functionally normal, these *RPLP2* pseudogenes are somewhat newly functional, and their population is characteristically expressed, which could contribute to their local adaptation to different environments and to the formation of some evolutionary traits, such as a larger body size [4]. In addition, these gene expansions are all driven by LTR transposons [8]. However, to date, little attention has been paid to other gene families that are associated with the genomic differentiation contributing to the local adaptation of this alpine butterfly species.

The Niemann–Pick C2 (*NPC2*) gene, characterized by the presence of the MD-2-related lipid recognition (ML) domain, was firstly reported and clarified as essential for lipid metabolism and cholesterol transport in vertebrates [10,11,12,13,14]. Nowadays, increasing evidence has shown that multiple *NPC2* genes are responsible for the regulation of olfactory communication, steroidogenesis, immune response, mating, and reproduction in some insect groups [15,16,17,18]. For example, eight *NPC2* genes (*NPC2a-h*) were identified in *Drosophila* for regulating steroidogenesis, growth, and molting by controlling sterol homeostasis and steroid biosynthesis [16]; furthermore, *NPC2e* and *NPC2a* were detected to bind bacterial cell wall components and function in immune signal pathways, respectively, in *Drosophila melanogaster* [18]. *NPC2* genes have also been shown to promote olfactory communication in the ant *Camponotus japonicas* [17], to help in the recognition of host plants in the honeybee *Apis cerana* [15], and in the mating and reproduction processes in the parasitoid wasp *Microplitis mediator* [19].

In this study, we attempted to conduct a comprehensive analysis of the *NPC2* gene family regarding their gene numbers, their correspondent protein physicochemical properties, and their gene structures and gene duplication mechanisms in 11 available butterfly species, focusing on the *Parnassius*, especially the *P. glacialis*. In addition, we analyzed the *NPC2* cis-acting elements to explore their regulatory mechanisms and the *NPC2* gene expression levels in different *P. glacialis* tissues through quantitative real-time PCR (qRT-PCR) to clarify their tissue-characteristic expressions. The results will deepen our understanding of the *Parnassius NPC2* gene family and provide some new insights into their local adaptation mechanisms.

## 2. Materials and Methods

### 2.1. The Identification of the NPC2 Gene Family

All genomic sequences and annotation files of 11 butterfly species, including the *P. glacialis*, were obtained based on two main criteria: phylogenetic representation across different butterfly families and genome availability. These species include *P. glacialis* and the *Parnassius apollo* [20] (GCA_907164705.1), the *P. orleans* [21] (GCA_029286625.1), the *Papilio machaon* (GCA_001298355.1), the *Papilio bianor* [22], the *Pieris napi* (GCA_905475465.1), the *Pieris rapae* (GCA_905147795.1), the *Fabriciana adippe* (GCA_905404265.1), the *Hesperia comma* (GCA_905404135.1), *Celastrina argiolus* (GCA_905187575.1), and *Heliconius erato* [23], which were obtained from the public NCBI (https://www.ncbi.nlm.nih.gov/genome, accessed on 14 August 2023), GigaDB (https://gigadb.org/dataset/100653, accessed on 14 August 2023) and LepBase (http://butterflygenome.org/?q=node/4/H.erato ltivitta v1.0, accessed on 14 August 2023) databases, respectively. The corresponding NPC2 protein sequences of *Drosophila melanogaster* (NP_731880.1) were downloaded from the GenBank of the NCBI (National Center for Biotechnology Information) database (https://www.ncbi.nlm.nih.gov/protein, accessed on 14 August 2023) for reference sequences; in order to identify the candidate *P. glacialis* NPC2 proteins, an analysis of a chromosome-level genome that was previously determined by our laboratory was performed using TBtools-II v2.154 [24] with the e-value threshold of 1 × 10^−5^. Additionally, the Hidden Markov Model (HMM) file of the *NPC2* DNA binding domain (PF02221) from the Pfam database (http://pfam.xfam.org/, accessed on 27 August 2023) was used to further identify the candidate *NPC2* gene sequences. Then, all of the above candidate gene sequences were verified using the NCBI-BLASTP (https://blast.ncbi.nlm.nih.gov/Blast.cgi?PROGRAM=blastp&PAGE_TYPE=BlastSearch&LINK_LOC=blasthome, accessed on 12 September 2023) and NCBI Batch-CDD search (https://structure.ncbi.nlm.nih.gov/Structure/cdd/wrpsb.cgi, accessed on 16 September 2023) methods. Finally, these verified genes of the *NPC2* family were renamed according to their locations on the chromosomes.

### 2.2. Analysis of Physicochemical Properties, Secondary Structure, and Subcellular Localization

The number of amino acids, molecular weight, isoelectric point, hydrophobic index, and other physicochemical properties of the above selected *NPC2* members were analyzed online using ExPASy (https://web.expasy.org/protparam, accessed on 28 September 2023). The secondary structures and the subcellular localizations of these NPC2 proteins were predicted using the online tools Network Protein Sequence analysis (https://npsa.lyon.inserm.fr/cgi-bin/npsa_automat.pl?page=/NPSA/npsa_sopma.html, accessed on 12 October 2023) and WoLF PSORT (https://wolfpsort.hgc.jp, accessed on 16 October 2023), respectively [25].

### 2.3. Analysis of Multiple Sequence Alignment and Phylogenetic

In order to explore the evolutionary relationship of these *NPC2* genes in butterflies, firstly, all *NPC2* sequences of the 11 butterfly species were retrieved from the genomic databases mentioned above; secondly multiple sequence alignment was performed, and the results were further processed using the Muscle program of the MEGA software (version 11) [26], and TrimAL 2.0 software [27], respectively; lastly, the best *NPC2* phylogenetic tree of the 11 butterfly species was reconstructed with the neighbor-joining method through 1000 bootstrap replicates using the MEGA software, and the evolutionary tree was visualized and beautified by the online software iTOL (https://itol.embl.de/, accessed on 19 August 2024) [28].

### 2.4. An Analysis of the Conserved Motifs, Gene Structures, and Characteristic Domains

The *NPC2* conserved motif was predicted using the MEME Suite v5.5.4 software (https://meme-suite.org/meme/, accessed on 2 April 2024) [29,30] with the E-value threshold of <0.05 and the following parameters: the minimum width (6 bp), maximum width (25 bp), and the maximum number of motifs (15). The *NPC2* exon–intron structure was retrieved from the GFF3 (General Feature Format 3) annotation file. The *NPC2* characteristic domain was identified using the NCBI website (https://structure.ncbi.nlm.nih.gov/Structure/cdd/wrpsb.cgi, accessed on 6 May 2024). Subsequently, the conserved motif, gene structure, and the characteristic domain of the *NPC2* genes were visualized using TBtools-II v2.154 [24].

### 2.5. An Analysis of the Chromosome Distribution, Collinearity, and Gene Duplication

To explore the chromosomal locations of *NPC2* genes of the three *Parnassius* butterfly species, the *NPC2* gene loci were retrieved from genomic files [24]. Both the gene duplication and collinearity analyses were performed using MCScanX in TBtools software with default parameters to investigate the evolutionary relationship of the *NPC2* genes in *Parnassius* species and two other Papilionidae species (*Pa. bianor* and *Pa. machaon*) [31,32,33]. Collinear gene pairs were defined based on at least five consecutive homologous gene pairs within syntenic blocks using an e-value threshold of 1 × 10^−5^. Gene duplication patterns were classified as follows [31]: (i) All genes were initially classified as ‘singletons’ and assigned gene ranks according to their order of appearance along chromosomes. (ii) Blastp results were evaluated, and the genes with blastp hits to other genes were re-labeled as ‘dispersed duplicates’. (iii) Genes with a rank difference of 1 were re-labeled as ‘tandem duplicates’. (iv) Genes with a rank difference of <20 were re-labeled as ‘proximal duplicates’. (v) The anchor genes in collinear blocks were re-labeled as ‘WGD/segmental’. All types of genomic transposable elements (TEs) were detected and annotated to explore their potential roles in shaping the *NPC2* duplication pattern using the HiTE v3.1.2 software (95% length coverage threshold) [34]. Gene family sequences were compared with annotated TE sequences using MEGA software (version 11) [26], and transposon frequencies within the 5 kb upstream and downstream regions of duplicated and non-duplicated genes were calculated and visualized using ggplot2 v2.1.0 to investigate the potential roles of TEs in gene duplication [35].

### 2.6. An Analysis of the Cis-Regulatory Elements

The 2 kb-sequence upstream and downstream of the *NPC2* start and end codon sites in the five Papilionidae species presented in this study were extracted and used for a cis-regulatory element analysis. Identification of cis-regulatory elements was performed using the plantCARE database (https://bioinformatics.psb.ugent.be/webtools/plantcare/html/, accessed on 22 November 2023) [36] to investigate the potential local adaptation mechanism through comparisons of *Parnassius* species with two other papilionids and through comparisons of *P. glacialis* with two other *Parnassius* species.

### 2.7. Genome Annotation and Kyoto Encyclopedia of Genes and Genome (KEGG) Pathway Enrichment

Gene annotation was performed using three methods: ab initio prediction, homology alignment, and RNA-seq support. Genescan v1.0 [37], Geneid v1.4 [38], SNAP v2013 [39], GlimmerHMM v3.04 [40], and Augustus v2.4 [41] tools were used for ab initio gene prediction. The homolog method was carried out using the software GeMoMa v1.3.1 [42] with default parameters. Functional annotations were obtained by searching the databases of NCBI-NR, Kyoto Encyclopedia of Genes and Genomes (KEGG) [43], Pfam [44], and SwissProt [45]. Gene functions for *P. glacialis* were assigned by aligning the protein sequences to the Kyoto Encyclopedia of Genes and Genome (KEGG) using Blastp (with the E-value threshold of ≤1 × 10^−5^), followed by a KEGG enrichment analysis of *PgNPC2* genes using TBtools, with a Benjamini–Hochberg-adjusted *p*-value < 0.05 as the significance threshold [46,47].

### 2.8. RNA Extraction and Quantitative RT-PCR

About ten *P. glacialis* individuals (adults and fourth instar larvae) were collected at Mountain Laoshan, Nanjing, China. After sample collection, the fresh samples were stored at −80 °C until RNA extraction. The total RNA from different tissues of larval (head and gonad) and adult (head, antennae, thorax muscle, and leg) samples were extracted using Trizol Reagent (Vazyme, Nanjing, China), and reversed cDNA was synthesized using the PrimeScript^TM^ 1st stand cDNA Synthesis Kit (Takara, Shanghai, China). The primers (Table S1) for *PgNPC2* gene amplification were synthesized by Shanghai Personal Biotechnology Co., Ltd. (Shanghai, China). The PCR reaction was performed using the following procedure: 95 °C for 5 min, 40 cycles of 95 °C for 15 s, and 60 °C for 30 s. The gene expression levels were analyzed using the comparative CT method (2^−∆∆CT^ method) with β-actin gene as the reference [48].

## 3. Results

### 3.1. Genome-Wide Identification and Physicochemical Properties of NPC2 Genes

In this study, a total of 30 *NPC2* genes were identified from chromosome-level genomes of 11 representative butterfly species (Table 1). Remarkably, ten *NPC2* genes were identified in *P. glacialis*, which were sequentially renamed from the *PgNPC2a* to the *PgNPC2j* according to their chromosomal locations, while only one or two *NPC2* genes were found in the other ten butterfly species. The sequence and physicochemical properties of these *NPC2* genes, including the protein length (from 103 aa to 192 aa), molecular weights (from 11.13 kDa to 21.71 kDa), theoretical isoelectric points (from 4.60 to 9.02), instability indices (from 27.47 to 58.35), lipolysis indices (from 76.67 to 114.78), and total mean hydrophilicity indices (from −0.280 to 0.526), are shown in Table 1. In light of these datasets, the amino acids, molecular weights, and instability indices of *Parnassius* were relatively larger than those of other butterfly species. Furthermore, all *PgNPC2* genes were shown to be located in the cytoplasm (Table S2), each harboring a typical signal peptide on their N-terminal ends.

### 3.2. A Phylogenetic Analysis of the NPC2 Gene Family Members in Representative Butterfly Genomes

**Figure 1:**
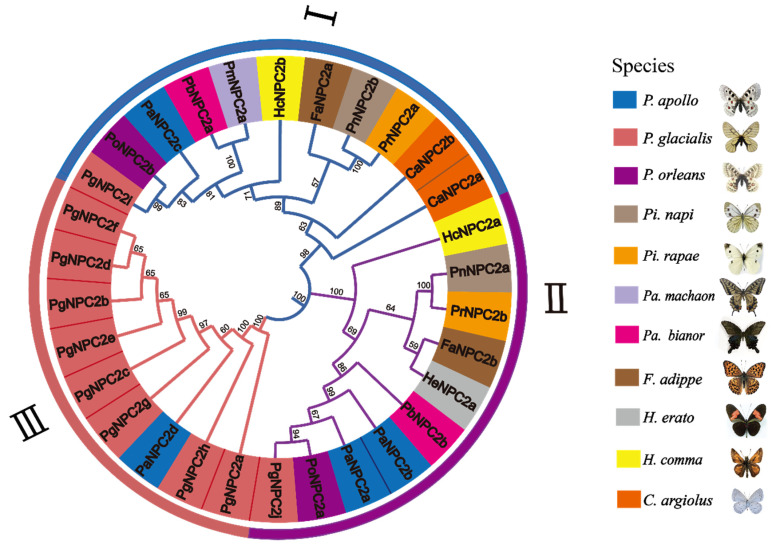
The phylogenetic relationships of the *NPC2* genes of the 11 representative butterfly species. The phylogenetic tree was reconstructed with the neighbor-joining (NJ) method through 1000 bootstrap replicates using MEGA 11.0.13. Different arcs indicate different subgroups (Cluster I–Cluster III). Different colors represent *NPC2* genes of different butterfly species.

All 30 *NPC2* sequences of 11 butterfly species were selected for a comparative analysis in this study to explore their evolutionary relationships (Figure S1). The reconstructed NJ tree showed that the *NPC2* genes of the butterflies in this study were divided into three subgroups (Figure 1), namely Cluster I (11 gene members), Cluster II (10 gene members), and Cluster III (9 gene members). In Cluster I, *PgNPC2i* from *P. glacialis*, *PoNPC2b* from *P. orleans*, and *PaNPC2c* from *P. apollo* shared the most common ancestor with high support values, and this small grouping was also found with other butterfly *NPC2* genes, while Cluster II contained *PgNPC2j* from *P. glacialis*, *PoNPC2a* from *P. orleans*, and *PaNPC2a* and *PaNPC2b* from *P. apollo*, which also shared a common ancestor with high support values. In contrast, Cluster III contained eight *NPC2* genes from *P. glacialis* and only one *NPC2* gene (*PaNPC2d*) from *P. apollo*. Thus, compared with other butterfly species that harbored only one or two *NPC2* genes, *P. glacialis* might have undergone a rapid expansion of *NPC2* genes.

### 3.3. Conserved Motifs, Characteristic Domains, and Gene Structures of NPC2 Genes

**Figure 2:**
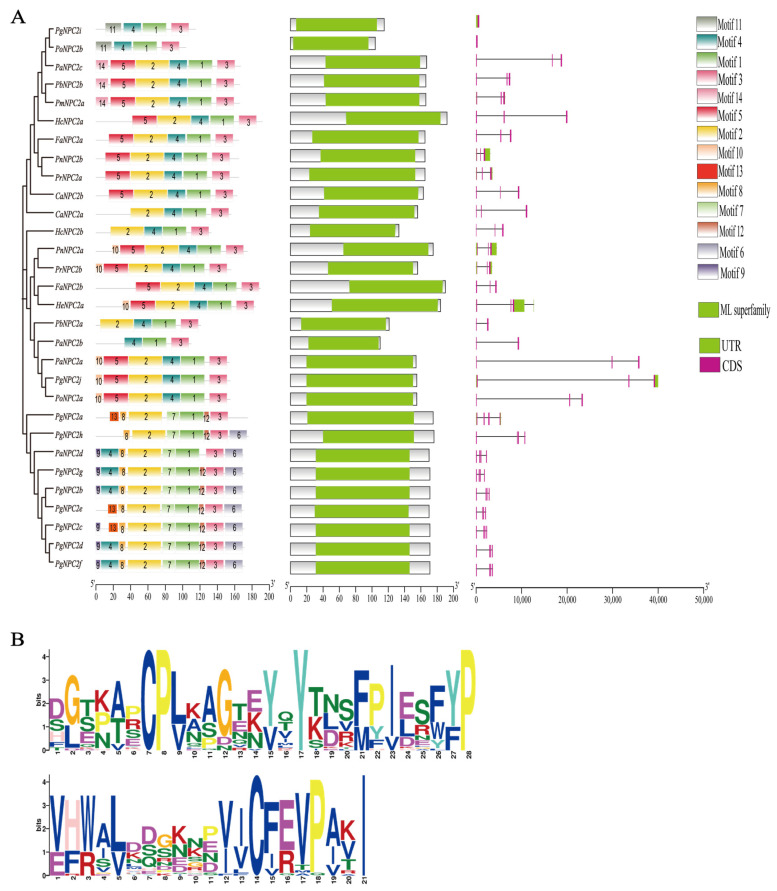
Structural characteristics of *NPC2* genes based on their phylogeny. (**A**) Left, schematic representation of conserved motifs of 30 *NPC2* proteins with different colored rectangles representing conserved motifs 1–14. (**A**) Middle, conserved domains, with green boxes representing MD-2-related lipid-recognition (ML) domain and gray boxes not containing domains. (**A**) Right, structure of exons (pink boxes) and introns (black lines) of *NPC2* genes, with green rectangles representing 3′ or 5′ end non-coding regions. (**B**) Conserved motifs: motif 1 and motif 3.

A comprehensive analysis of the *NPC2* properties (Figure 2A and Figure S3) showed that in total, 14 conserved motifs (motifs 1–14) were identified among the 30 *NPC2* gene members using the online MEME and TBtools-II software, with the lengths ranging from 6 to 50 bp (Figure 2A). Interestingly, all *NPC2* genes contained motifs 1 and 3 (Figure 2B), which are likely key components involved in functional specificity, and they were found in Clusters I and II along with motif 4, and Cluster III contained motifs 1, 2, 3, and 8.

A conserved domain analysis showed that only one conserved domain (MD-2-related lipid-recognition, ML) existed in all 30 *NPC2* members of the butterflies (Figure 2A). The intron–exon structure analysis showed that all *NPC2* gene members of the same cluster exhibited similar structures, that is, they shared identical exon numbers (Figure 2A). Interestingly, *PgNPC2j*, a member of Clusters I and II, contains three exons and two exceptionally long introns, while *PgNPC2i* contains only one exon and lacks introns; however, the *PgNPC2* genes from Cluster III all contain four exons and three introns (Figure 2A).

### 3.4. Chromosomal Location, Gene Duplication, and Collinearity Analysis

**Figure 3:**
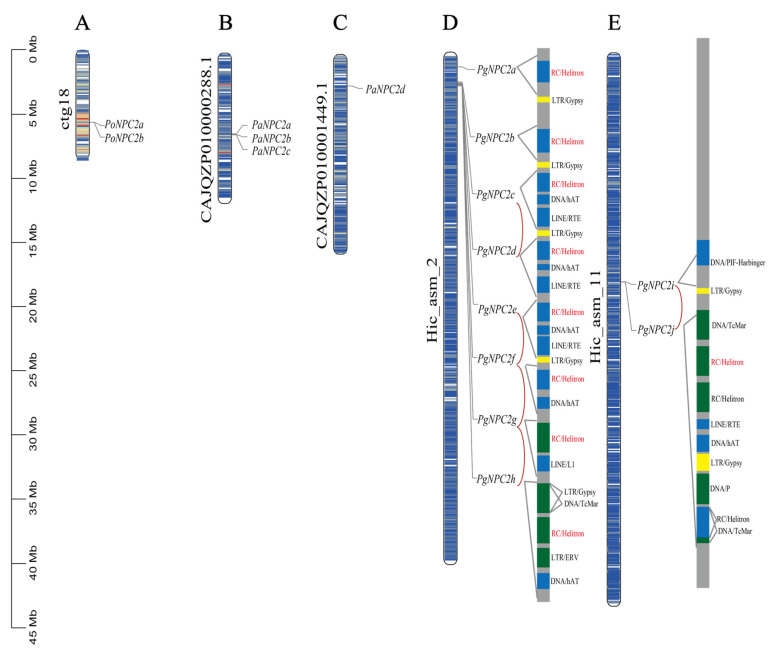
Chromosomal locations of the *P. orleans*, *P. apollo*, and *P. glacialis NPC2* genes. (**A**) *P. orlens* chromosomal contig18. (**B**,**C**) *P. apollo* chromosomal contigCAJQZP010000288.1 and contigCAJQZP010001449.1. (**D**,**E**) *P. glacialis* chromosomes Hic-asm-2 and Hic-asm-11. Tandem duplicated gene pairs of *P. glacialis* are shown in the red arc. The green boxes represent complete transposons, the blue boxes represent partial transposons, and the yellow boxes represent LTR/Gypsy transposons.

A chromosomal location analysis showed that the *NPC2* genes of the *Parnassius* butterfly species were all located on one or two chromosomes, and for *P. glacialis*, its 10 *PgNPC2* genes were unevenly located on two chromosomes: the majority of *PgNPC2* genes (eight genes) were on the chromosome Hic-asm-2, while two *PgNPC2* genes were on the chromosome Hic-asm-11 (Figure 3).

A gene duplication analysis showed that a total of five tandem duplication *NPC2* gene pairs were located on chromosomes Hic-asm-2 (*PgNPC2c/PgNPC2d*, *PgNPC2e/PgNPC2f*, *PgNPC2f/PgNPC2g*, and *PgNPC2g/PgNPC2h*) and Hic-asm-11 (*PgNPC2i/PgNPC2j*) (Figure 3). Additionally, *PgNPC2a* and *PgNPC2b* on Hic-asm-2 are proximal duplicates, suggesting that a complex pattern of gene duplication events existed in this genomic region. A gene duplication mechanism analysis showed that six major types of transposable elements, including DNA/hAT, RC/Helitron, LINE/RTE, LINE/L1, LTR/Pao, and LTR/Gypsy, were responsible for these duplication events (Table 2 and Table S4), and furthermore, within these transposon-mediated genes, *PgNPC2g*, *PgNPC2h*, and *PgNPC2j* were fully and partially transposon mediated, while the other seven genes were partially transposon-derived sequences (Figure 3). For the Hic-asm-2 chromosome, the *PgNPC2* genes all contained the RC/Helitron and DNA/hAT transposons, with the exception of *PgNPC2a* and *PgNPC2g* lacking DNA/hAT. Additionally, most *PgNPC2* genes were 1000 bp upstream or downstream and linked with the LTR/Gypsy retrotransposons, while *PgNPC2c*, *PgNPC2d*, *PgNPC2e*, and *PgNPC2j* were all derived from the insertion of LINE/RTE transposons. To investigate the potential role of TEs in mediating gene duplication events, we compared the sequences of gene family members with annotated and identified TE sequences. We found that an RC/Helitron transposon present in all *PgNPC2* genes on the chromosome Hic-asm-2 is highly similar to the RC/Helitron transposon in the *PgNPC2j* gene on the chromosome Hic-asm-11 (Figure S2). In addition, by calculating the frequencies of transposons within the 5 kb upstream and downstream regions of duplicated and non-duplicated genes, it was found that the frequency of transposons was significantly higher near the duplicated genes, suggesting that a potential association exists between TEs and gene duplication (Figure S3).

**Figure 4:**
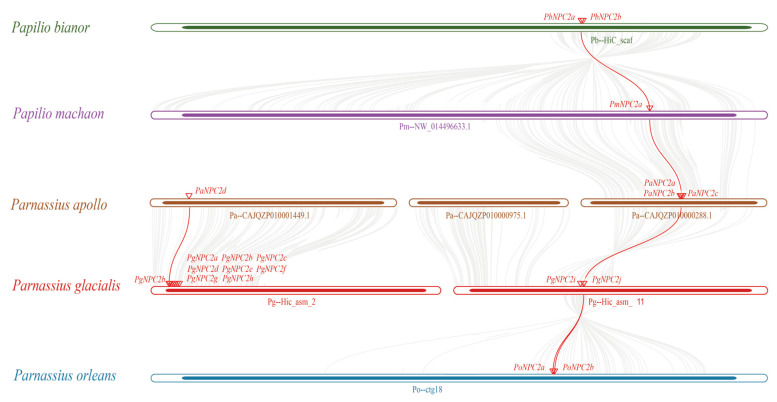
A plot of interspecific collinearity among *P. glacialis*, *Pa. bianor*, *Pa. machaon*, *P. apollo*, *P. glacialis*, and *P. orleans*. The gene pairs are highlighted by the red lines, with duplicated genes shown nearby. The gray lines indicate collinear blocks between chromosomes or scaffolds of different species.

The collinear relationships between 10 *PgNPC2* genes and 9 other papilionid *NPC2* genes are shown in Figure 4. Gene pairs (*PbNPC2b/PgNPC2j*, *PmNPC2b/PgNPC2j*, *PaNPC2a/PgNPC2j*, and *PoNPC2b/PgNPC2j*) were located on the chromosomal locations Pb-HIC_scaffold_12, Pm-NW_014496633.1, Pa-CAJQZP010000288.1, Pg-HIC_asm_11, and Po-ctg18 of five species (*Pa. bianor*, *Pa. machaon*, *P. apollo*, *P. glacialis*, and *P. orleans*), respectively; another one (*PaNPC2d/PgNPC2b*) was located on the chromosomes Pa-CAJQZP010001449.1 and Pg-HIC_asm_2 of *P. glacialis* and *P. apollo*. Obviously, there were four unique tandem repeats of *P. glacialis NPC2* genes (*PgNPC2c/PgNPC2d*, *PgNPC2e/PgNPC2f*, *PgNPC2f/PgNPC2g* and *PgNPC2g/PgNPC2h*) that were likely to be newly duplicated.

### 3.5. Analysis of Cis-Regulatory Elements of NPC2 Genes

**Figure 5:**
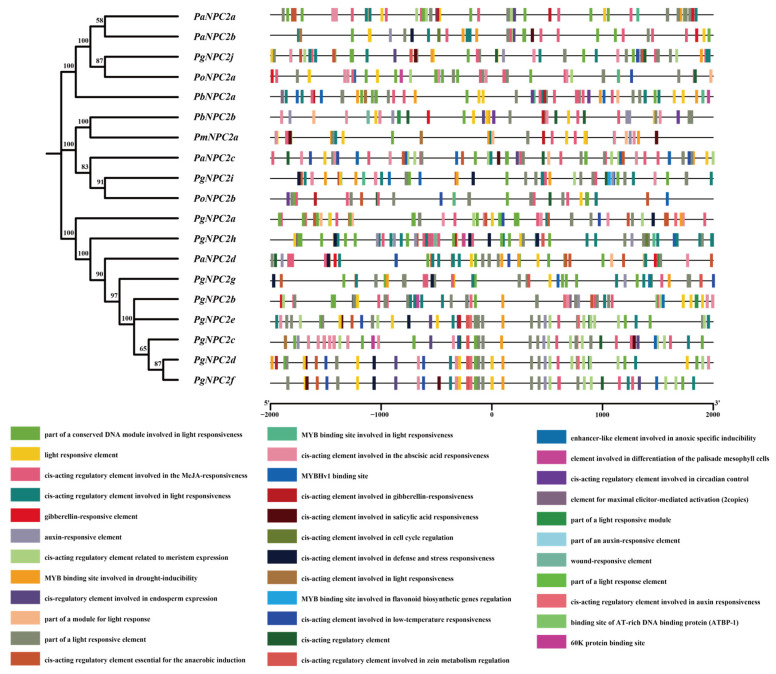
Cis-regulatory elements (CREs) of the papilionid *NPC2* genes. The black lines represent a 2 kb sequence upstream and downstream of the start and stop codon sites of *NPC2* genes, and the different colored boxes correspond to different CREs.

Different cis-regulatory elements within the gene promoter can result in distinct gene expression patterns [49]. Our analysis of the cis-acting elements showed that all five butterfly species contained light-responsive cis-acting elements; the cis-acting elements related to anaerobic induction, low-temperature acclimatization, and stress responses were especially rich in the *Parnassius* genomes, and other cis-acting elements, such as the binding site of AT-rich DNA binding protein (ATBP-1) and a wound-responsive element, were detected in *P. glacialis* (Figure 5).

### 3.6. The KEGG Enrichment Analysis for PgNPC2 Genes of P. glacialis

**Figure 6:**
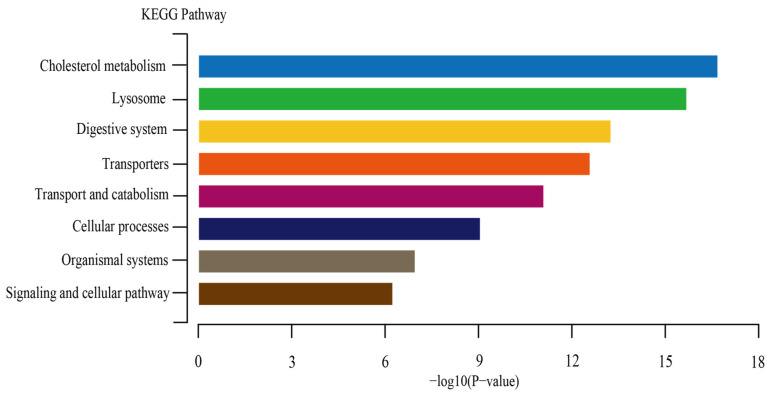
Enriched KEGG pathways for the *PgNPC2* genes in the *P. glacialis* genome. Each bar represents a different biological pathway. The *x*-axis represents the Benjamini–Hochberg-adjusted *p*-value, where the smaller the *p*-value, the higher the enrichment of the pathway. The *y*-axis represents the pathways identified in the KEGG enrichment analysis.

The KEGG enrichment analysis results indicate that the *PgNPC2* gene of *P. glacialis* was significantly enriched in pathways related to cholesterol metabolism, lysosomal metabolism, digestive system, transport and catabolism, cellular processes, organismal systems, and signaling and cellular pathways. Among these pathways, the cholesterol metabolism harbored the highest significance (−Log10(*p*-value) ~18), while the signaling and cellular pathways showed the lowest significance (−Log10(*p*-value) > 6) (Figure 6, Table S5).

### 3.7. The Variance of NPC2 Gene Expression Levels in Different Tissues of P. glacialis

**Figure 7:**
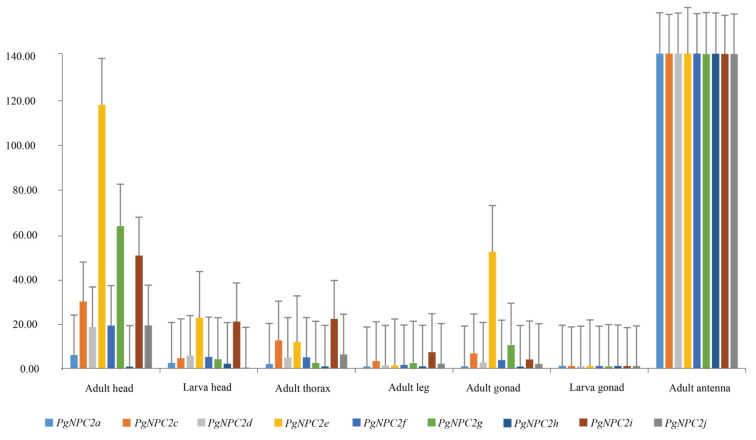
The expression levels of the nine *PgNPC2* genes in different *P. glacialis* tissues. The *x*-axis represents different tissues, and the *y*-axis represents the gene expression levels (relative expression, 2^−ΔΔCt^). The *y*-axis is truncated to better visualize differences in expression levels. The bar graph is enhanced with error bars to facilitate a more intuitive comparison of expression differences, and the different colors of the bars represent different *NPC2* genes.

All ten *PgNPC2* genes in the *P. glacialis* genome were used for qRT-PCR verification, and the results indicate that all *PgNPC2* genes were expressed at relatively low levels in the thoraxes, legs, and gonads of the adults, as well as in the heads and gonads of the larvae; however, nine of the ten *PgNPC2* genes were highly expressed in the antennae and moderately expressed in the heads of the adults. These facts indicate that the adult *P. glacialis* antennae and heads were likely to play a key role in the local adaptation of olfactory communication, host plant recognition, and reproduction through high-level *PgNPC2* expression (Figure 7 and Table S4).

## 4. Discussion

In this study, a total of 10 genes of the *NPC2* gene family in *P. glacialis* were identified for the first time through a genome-wide analysis of the butterflies available. All *PgNPC2* genes contain a highly conserved MD-2-related lipid-recognition (ML) domain and six cysteines, which is consistent with other insect groups [15,19]. However, our analysis revealed that, compared to other butterfly species, the *P. glacialis* had a significant expansion of *NPC2* genes, which could have been driven by tandem duplications mediated by transposon insertions, causing motif structure changes accompanied by intron gains or losses [50].

Previous studies showed that as the distributional elevation increased, the genome size of the *Parnassius* butterflies generally decreased, as in case of the three *Parnassius* species (*P. orleans* (1.23 Gb), *P. apollo* (~1.39), and *P. glacialis* (~1.35 Gb)) in this study. Our phylogenetic and collinearity analyses revealed that the *Parnassius* species, especially *P. apollo* and *P. glacialis*, harbored relatively larger amounts of *NPC2* genes (four and ten, respectively) than other butterfly groups (one or two). Phylogenetically, the eight *PgNPC2* genes and *PaNPC2d* in Cluster III shared the nearest common ancestor, with the former gene clusters being shaped by rapid tandem duplications [4,8]. These expanded genes in *Parnassius* may have contributed to variations in lipid or cholesterol metabolism (Figure 6), potentially providing a selective advantage at the relatively low-altitude habitats in which the oxygen content and temperature are relatively higher; in addition, the *NPC2* expansions might also be somewhat attributed to the genome size increases that provided genetic make-ups for local adaptation to distinct ecological niches [8,32].

Gene duplication was shown to be one of the key factors in shaping genome diversity for adaptive evolution [48,51,52,53,54,55,56]. For the expansion of gene family, tandem repeat and TE-mediated duplication are the two main mechanisms [55,56]. A large amount of *PgNPC2* tandem duplications in *P. glacialis* were also found in this study, verifying the fact that the tandem repeat of functional genes is probably associated with local adaptation, as shown in previous studies. For example, P450 tandem duplications were shown to be linked to the biosynthesis of defense compounds, pigments, antioxidants, and detoxification processes, as well as to adaptation to environmental changes, in coffee, tomato, and other Solanaceae plants [55,57]. Recently, TEs, especially LTR-TEs were identified as the major drivers of genome evolutionary adaptation [58,59], and other TEs, such as the Helitron, were also shown to probably facilitate gene duplication in maize and bats by capturing roll-loop-mediated gene fragments [60,61]. Our study revealed that the *P. glacialis* Helitron transposons were responsible for the duplication of *PgNPC2* genes on the Hic-asm-2 chromosome, while the LINE and LTR retrotransposons likely contributed to intronless pseudogenes, a few of which, such as the *PgNPC2i*, showed a detectable level of expressions through qRT-PCR (Figure 7), as found in previous studies [62]; however, further functional assays are required to confirm their physiological roles.

Our cis-acting element analysis showed that the five papilionid butterfly species all contained light-responsive cis-acting elements that were associated with circadian rhythms and behaviors of forage and migration [63]; in addition, the *Parnassius* species was especially enriched with elements related to anaerobic induction, low-temperature acclimatization, and environmental stress responses, which were likely to be responsible for their adaptation to high-altitude environments. Furthermore, the ATBP-1 binding sites and wound-responsive elements found only in *P. glacialis* might be involved in wound response and immune defense to pathogen invasion and stress in relatively low-altitude mountain areas [64].

Additionally, our qRT-PCR analysis revealed that a total of nine *NPC2* genes were highly expressed in the head, especially in the antennae; however, these *NPC2* genes were lowly expressed in other tissues of the *P. glacialis*. As far as we know, *NPC2* genes are mainly involved in olfactory communication, which influence the habitat selection and mate recognition of insects [15,17,19], and thus, the *P. glacialis* antennae and other sensory organs on the head might play a potential role in the species’ ‘out of Qinghai-Tibet Plateau’ dispersal from a northeastern direction. However, further functional tests are also needed to confirm the specific roles of these genes in olfactory communication and dispersal adaptation.

## 5. Conclusions

The study analyzed the *NPC2* gene family in the genomes of 11 representative butterfly species, including 3 *Parnassius* species, and systematically examined the gene structures and phylogenetic relationships with a focus on the gene duplication and expression patterns of *P. glacialis*. The results indicate that the *NPC2* genes of *P. glacialis* had a significant expansion driven by tandem duplications mediated by transposon insertions. Furthermore, the qRT-PCR analysis showed that *NPC2* genes were highly expressed in the antenna, followed by the head, with low expression in other tissues. This study provides a comprehensive overview of the *NPC2* gene family of *P. glacialis* and offers new insights into the local adaptation mechanisms of this alpine butterfly species.

## Figures and Tables

**Table 1:** Physicochemical properties of *NPC2* genes identified in this study.

Species	Gene Name	Gene ID	Amino Acid Number/aa	Molecular Weight /Da	Theoretical Isoelectric	Instability	Stability	Lipolysis Indices	Total Mean Hydrophilic Value
*P. apollo*	*PaNPC2a*	Papo004992.1	154	17,122.9	5.73	50.96	unstable	99.29	−0.023
	*PaNPC2b*	Papo004994.1	110	12,421.3	5.38	53.96	unstable	87.64	−0.093
	*PaNPC2c*	Papo004995.1	167	18,299.3	8.63	38.97	stable	93.89	0.116
	*PaNPC2d*	Papo023738.1	170	18,628.9	8.97	37.31	stable	98.12	0.094
*P. glacialis*	*PgNPC2a*	evm.model.CTG_193.50	174	19,187.2	4.6	49.94	unstable	98.45	0.326
	*PgNPC2b*	evm.model.CTG_193.68	170	18,719.6	8.28	40.11	unstable	92.29	0.039
	*PgNPC2c*	evm.model.CTG_193.70	170	18,671.6	8.63	40.36	unstable	94.59	0.066
	*PgNPC2d*	evm.model.CTG_193.71	170	18,699.6	8.28	39.11	stable	94.59	0.05
	*PgNPC2e*	evm.model.CTG_54.223	169	18,632.5	8.28	42.1	unstable	92.84	0.027
	*PgNPC2f*	evm.model.CTG_54.222	170	18,699.6	8.28	39.11	stable	94.59	0.05
	*PgNPC2g*	evm.model.CTG_54.221	170	18,607.4	8.27	48.95	unstable	86	−0.002
	*PgNPC2h*	evm.model.CTG_54.220	175	19,299.3	9.02	41.31	unstable	89.09	−0.11
	*PgNPC2i*	evm.model.CTG_162.121	114	12,533.5	5.17	58.35	unstable	105.96	0.32
	*PgNPC2j*	evm.model.CTG_162.120	154	16,928.6	5.63	51.2	unstable	94.87	−0.008
*P. orleans*	*PoNPC2a*	evm.model.ctg18.150	154	16,968.6	6.07	39.09	stable	92.34	−0.103
	*PoNPC2b*	evm.model.ctg18.151	103	11,132.8	5.45	56.63	unstable	97.38	0.256
*Pi. napi*	*PnNPC2a*	Pnap002095.1	175	19,309.5	8.03	42.62	unstable	86.17	−0.065
	*PnNPC2b*	Pnap002017.1	165	18,105	8.39	37.3	stable	81.52	−0.108
*Pi. rapae*	*PrNPC2a*	Prap002064.1	165	18,129	7.5	42.05	unstable	86.24	−0.127
	*PrNPC2b*	Prap002333.1	156	17,008	8.07	27.47	stable	99.23	0.053
*Pa. machaon*	*PmNPC2a*	Pmac012693.1	166	18,221.1	6.72	33.02	stable	94.4	0.051
*Pa. bianor*	*PbNPC2a*	Pbia004755.1	121	13,646.9	8.58	45.37	unstable	82.07	−0.086
	*PbNPC2b*	Pbia004754.1	166	18,211	6.71	33.5	stable	91.51	0.017
*F. adippe*	*FaNPC2a*	Fadi023545.1	165	18,367.3	8.21	35.33	stable	92.67	−0.096
	*FaNPC2b*	Fadi023544.1	190	21,707.5	8.9	50.38	unstable	89.11	−0.056
*H. erato*	*HeNPC2a*	Hera002910.1	157	17,543.6	5.26	38.23	stable	114.78	0.526
*H. comma*	*HcNPC2a*	Hcom020357.1	192	21,082.2	8.8	38.06	stable	76.67	−0.084
	*HcNPC2b*	Hcom020358.1	133	14,664	7.62	38.45	stable	82.03	−0.095
*C. argiolus*	*CaNPC2a*	Carg014702.1	156	17,534.2	8.48	38.82	stable	85.51	−0.28
	*CaNPC2b*	Carg004219.1	163	17,910.6	6.41	29.3	stable	89.02	−0.064

**Table 2:** Major types of transposable elements in *PgNPC2* genes of *P. glacialis*.

Gene ID	Chromosome	Position Relation	Start	End	Transposon Type
*PgNPC2a*	Hic_asm_2	gene contains TE	1335330	1335554	RC/Helitron
*PgNPC2a*	Hic_asm_2	gene close to TE	1338311	1338500	LTR/Gypsy
*PgNPC2b*	Hic_asm_2	gene contains TE	2517825	2518233	RC/Helitron
*PgNPC2b*	Hic_asm_2	gene contains TE	2519529	2519773	LINE/RTE
*PgNPC2b*	Hic_asm_2	gene close to TE	2520625	2520760	LTR/Gypsy
*PgNPC2c*	Hic_asm_2	gene contains TE	2592201	2592612	RC/Helitron
*PgNPC2c*	Hic_asm_2	gene contains TE	2593177	2593421	DNA/hAT
*PgNPC2c*	Hic_asm_2	gene contains TE	2593755	2594059	LINE/RTE
*PgNPC2c*	Hic_asm_2	gene close to TE	2594490	2594684	LTR/Gypsy
*PgNPC2d*	Hic_asm_2	gene contains TE	2609212	2609717	RC/Helitron
*PgNPC2d*	Hic_asm_2	gene contains TE	2610207	2610263	DNA/hAT
*PgNPC2d*	Hic_asm_2	gene contains TE	2612034	2612351	LINE/RTE
*PgNPC2e*	Hic_asm_2	gene contains TE	2660198	2660703	RC/Helitron
*PgNPC2e*	Hic_asm_2	gene contains TE	2660910	2661154	DNA/hAT
*PgNPC2e*	Hic_asm_2	gene contains TE	2661489	2661741	LINE/RTE
*PgNPC2e*	Hic_asm_2	gene close to TE	2662201	2662395	LTR/Gypsy
*PgNPC2f*	Hic_asm_2	gene contains TE	2701489	2701971	RC/Helitron
*PgNPC2f*	Hic_asm_2	gene contains TE	2702201	2702445	DNA/hAT
*PgNPC2f*	Hic_asm_2	gene contains TE	2704393	2704618	LINE/RTE
*PgNPC2g*	Hic_asm_2	gene contains TE	2712171	2712725	RC/Helitron
*PgNPC2g*	Hic_asm_2	gene contains TE	2712845	2713124	LINE/L1
*PgNPC2h*	Hic_asm_2	gene contains TE	2722118	2722514	LTR/Gypsy
*PgNPC2h*	Hic_asm_2	gene contains TE	2723163	2723709	RC/Helitron
*PgNPC2h*	Hic_asm_2	gene contains TE	2729218	2729589	LTR/ERV
*PgNPC2h*	Hic_asm_2	gene contains TE	2729769	2730136	DNA/hAT
*PgNPC2i*	Hic_asm_11	gene overlaps TE	17857018	17857217	DNA/PIF-Harbinger
*PgNPC2i*	Hic_asm_11	gene close to TE	17858425	17858553	LTR/Gypsy
*PgNPC2j*	Hic_asm_11	gene contains TE	17875148	17878336	LINE/RTE
*PgNPC2j*	Hic_asm_11	gene contains TE	17866674	17867025	DNA/TcMar
*PgNPC2j*	Hic_asm_11	gene contains TE	17871065	17871268	RC/Helitron
*PgNPC2j*	Hic_asm_11	gene contains TE	17873433	17875156	RC/Helitron
*PgNPC2j*	Hic_asm_11	gene contains TE	17879907	17880988	DNA/hAT
*PgNPC2j*	Hic_asm_11	gene contains TE	17891108	17893662	DNA/P
*PgNPC2j*	Hic_asm_11	gene contains TE	17894246	17894618	DNA/TcMar

## Data Availability

The genome data of *P. glacialis* were deposited into GenBank with the BioProject number PRJNA893814.

## Supplementary Materials

The following supporting information can be downloaded at www.mdpi.com/article/10.3390/genes16030249/s1, Figure S1: Alignment of amino acid sequences of *NPC2* proteins from 11 butterfly species; Box represents conserved domain of *NPC2* gene family, with black and gray shaded areas representing conserved sites; Figure S2: Transposon sequence alignment in the *PgNPC2* genes of *P. glacialis*. The black and gray areas represent highly conserved transposon sites in different *PgNPC2* genes; Figure S3: Transposon frequencies in the upstream and downstream 5 kb regions of duplicated genes and non-duplicated genes of the *NPC2* genes in the genome of *P. glacialis*. The vertical axis represents the frequency of transposon occurrence, with the red box plot representing the downstream region of genes and the blue box plot representing the upstream region of genes; Table S1: Primer information of the *PgNPC2* genes; Table S2: Secondary structure and subcellular localization of *PgNPC2* genes of *P. glacialis*; Table S3: Information of identified motifs; Table S4: Transposable element information in *PgNPC2* genes of *P. glacialis*; Table S5: Information about KEGG pathways for *PgNPC2* genes of *P. glacialis*; Table S6: Sampling information about *P. glacialis* for qRT-PCR.
