# Supplementary material for: Genome-Wide Identification, Gene Duplication, and Expression Pattern of NPC2 Gene Family in Parnassius glacialis
Source: Genes (Basel). 2025 Feb 21;16(3):249. doi: 10.3390/genes16030249 (PMC11942102; doi:10.3390/genes16030249)
Supplement: Supplementary file 1 [file genes-16-00249-s001.zip › genes-3471625-supplementary/Supplementary_Figure.pdf]

Supplementary Materials

Genome-wide Identification, Gene Duplication and Expression Pattern of the *NPC2* gene family in *Parnassius glacialis*

Supplementary Figure S1. Alignment of amino acid sequences of *NPC2* proteins from 11 butterfly species. The Box represent the conserved domain of the *NPC2* gene family, with black and gray shaded areas representing conserved sites.

Supplementary Figure S2. Transposons sequence alignment in the *PgNPC2* genes of *P. glacialis*. The black and gray areas represent highly conserved transposon sites in different *PgNPC2* genes.

Supplementary Figure S3. Transposon frequencies in the upstream and downstream 5 kb regions of duplicated genes and non-duplicated genes of the *NPC2* genes in the genome of *P. glacialis*. The vertical axis represents the frequency of transposon occurrence, with the red box plot representing the downstream region of genes and the blue box plot representing the upstream region of genes.

Supplementary Table S1. Primer information of the *PgNPC2* genes.

Supplementary Table S2. Secondary structure and subcellular localization of *PgNPC2* genes of *P. glacialis.*

Supplementary Table S3. Information of identified motifs.

Supplementary Table S4. Transposable elements information in *PgNPC2* genes of *P. glacialis.*

Supplementary Table S5. The information of KEGG pathways for *PgNPC2* genes of *P. glacialis.*

Supplementary Table S6. Sampling information of *P. glacialis* for qRT-PCR.

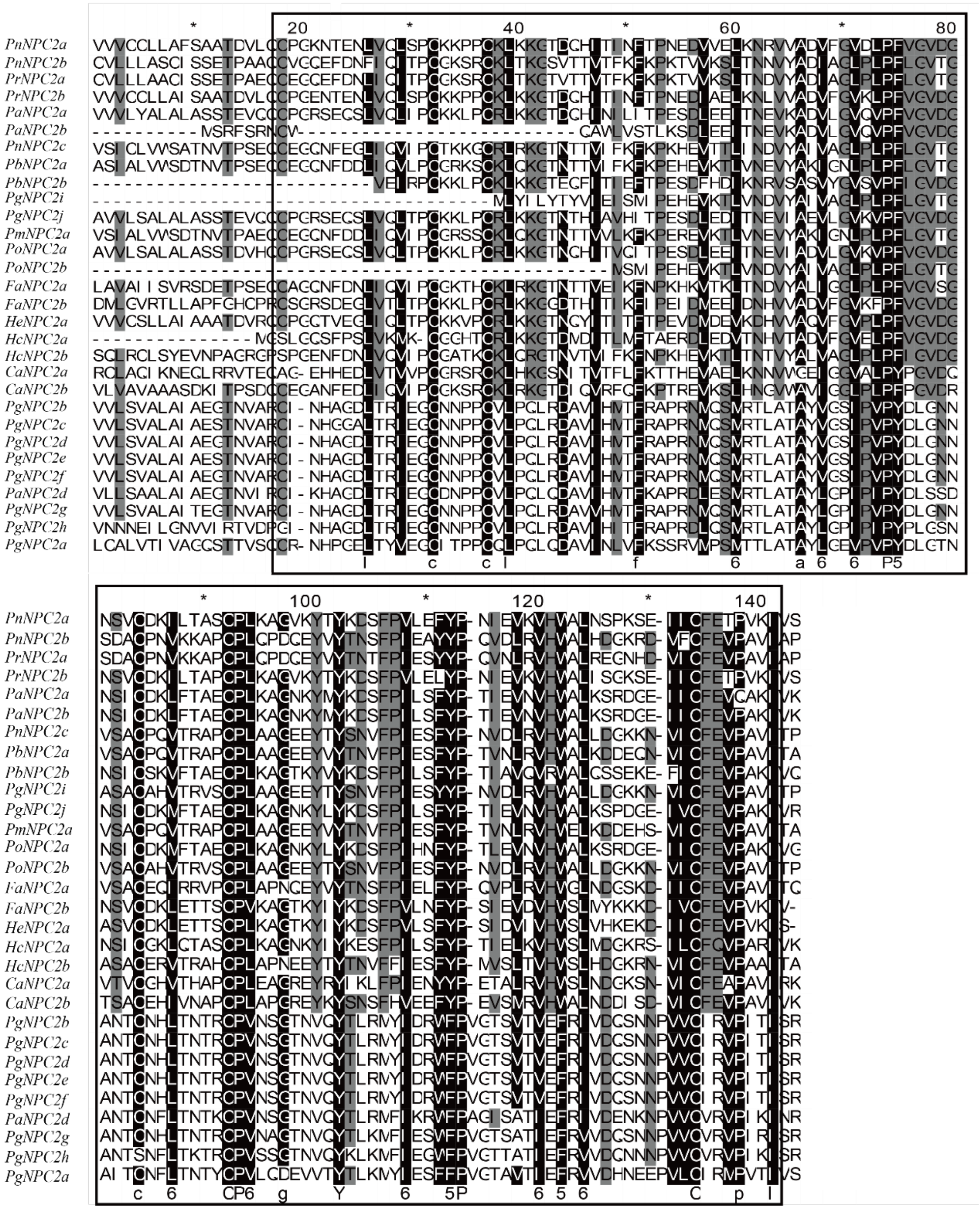

Figure S1. Alignment of amino acid sequences of *NPC2* proteins from 11 butterfly species. The Box represent the conserved domain of the *NPC2* gene family, with black and gray shaded areas representing conserved sites.

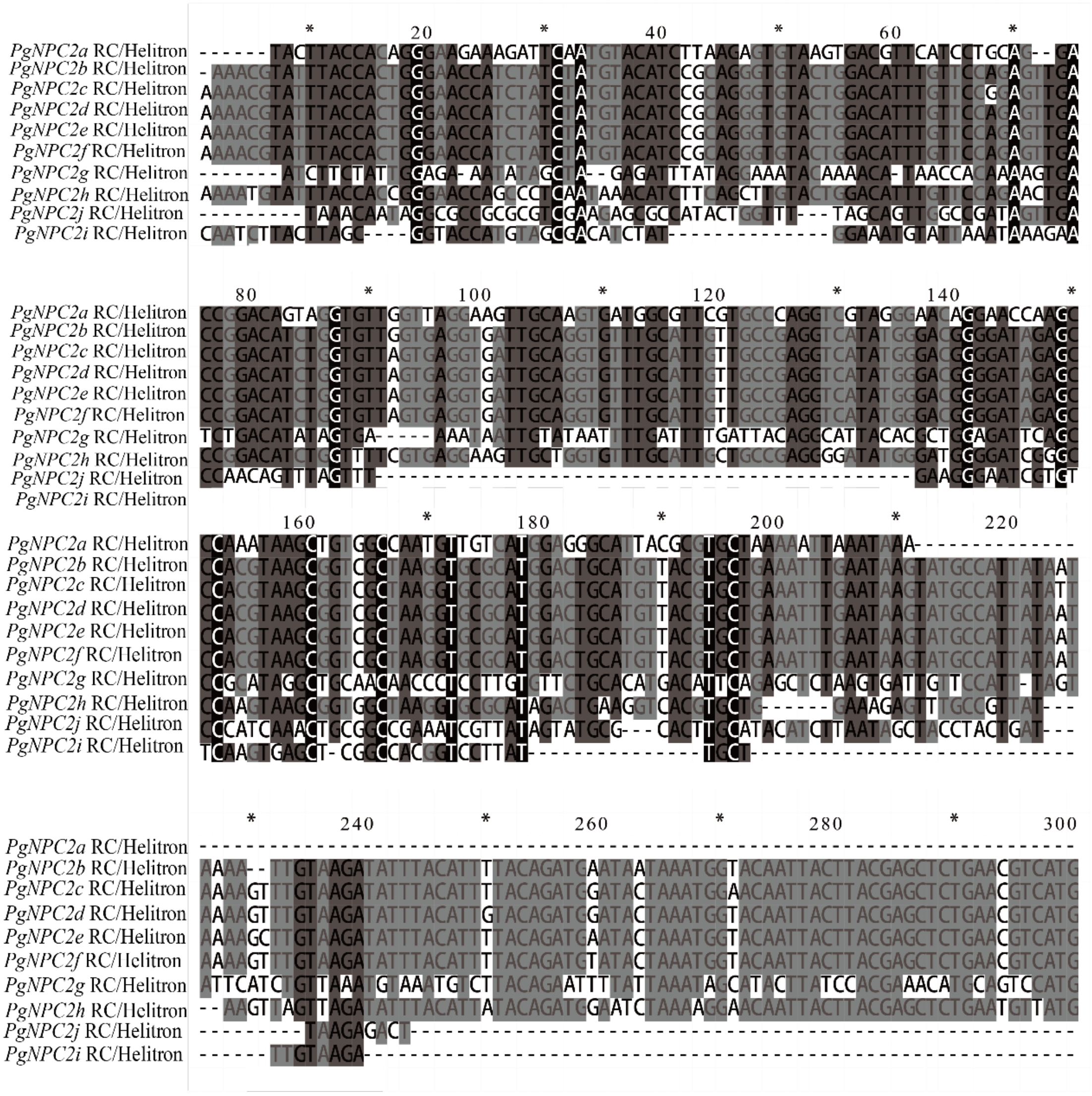

Figure S2. Transposons sequence alignment in the *PgNPC2* genes of *P. glacialis.* The black and gray areas represent highly conserved transposon sites in different *PgNPC2* genes.

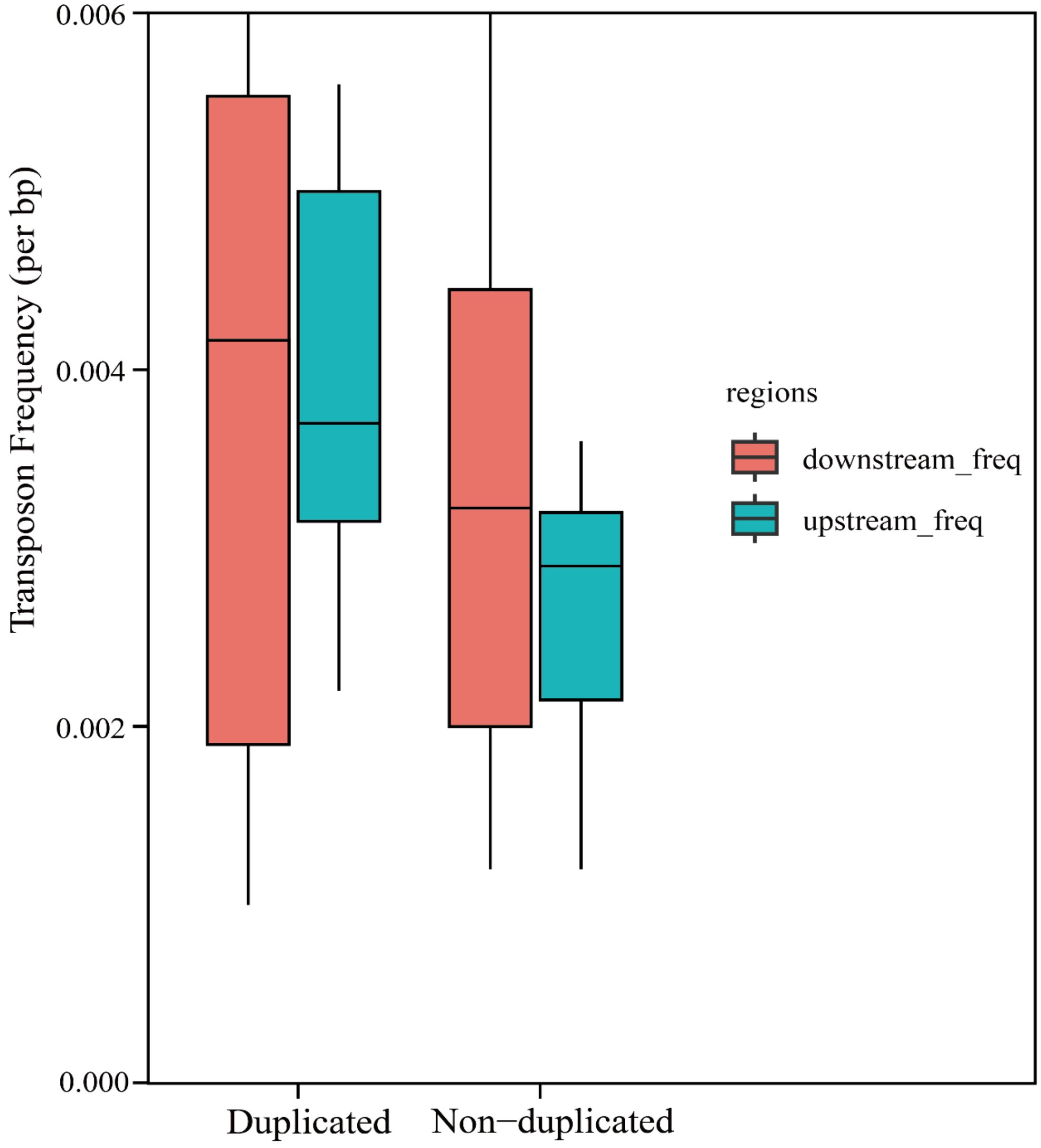

Supplementary Figure S3. Transposon frequencies in the upstream and downstream 5 kb regions of duplicated genes and non-duplicated genes of the *NPC2* genes in the genome of *P. glacialis*. The vertical axis represents the frequency of transposon occurrence, with the red box plot representing the downstream region of genes and the blue box plot representing the upstream region of genes.
